# Parent-Reported Use of Pediatric Primary Care Telemedicine: Survey Study

**DOI:** 10.2196/42892

**Published:** 2023-02-09

**Authors:** Kristin N Ray, Samuel R Wittman, Sarah Burns, Tran T Doan, Kelsey A Schweiberger, Jonathan G Yabes, Janel Hanmer, Tamar Krishnamurti

**Affiliations:** 1 Department of Pediatrics University of Pittsburgh Pittsburgh, PA United States; 2 Department of Medicine University of Pittsburgh Pittsburgh, PA United States

**Keywords:** telehealth, telemedicine, pediatrics, primary care

## Abstract

**Background:**

Telemedicine delivered from primary care practices became widely available for children during the COVID-19 pandemic.

**Objective:**

Focusing on children with a usual source of care, we aimed to examine factors associated with use of primary care telemedicine.

**Methods:**

In February 2022, we surveyed parents of children aged ≤17 years on the AmeriSpeak panel, a probability-based panel of representative US households, about their children’s telemedicine use. We first compared sociodemographic factors among respondents who did and did not report a usual source of care for their children. Among those reporting a usual source of care, we used Rao-Scott *F* tests to examine factors associated with parent-reported use versus nonuse of primary care telemedicine for their children.

**Results:**

Of 1206 respondents, 1054 reported a usual source of care for their children. Of these respondents, 301 of 1054 (weighted percentage 28%) reported primary care telemedicine visits for their children. Factors associated with primary care telemedicine use versus nonuse included having a child with a chronic medical condition (87/301, weighted percentage 27% vs 113/753, 15%, respectively; *P*=.002), metropolitan residence (262/301, weighted percentage 88% vs 598/753, 78%, respectively; *P*=.004), greater internet connectivity concerns (60/301, weighted percentage 24% vs 116/753, 16%, respectively; *P*=.05), and greater health literacy (285/301, weighted percentage 96% vs 693/753, 91%, respectively; *P*=.005).

**Conclusions:**

In a national sample of respondents with a usual source of care for their children, approximately one-quarter reported use of primary care telemedicine for their children as of 2022. Equitable access to primary care telemedicine may be enhanced by promoting access to primary care, sustaining payment for primary care telemedicine, addressing barriers in nonmetropolitan practices, and designing for lower health-literacy populations.

## Introduction

In 2020, payer and regulatory changes facilitated delivery of primary care telemedicine to pediatric patients. While models for delivery of subspecialty care and commercial direct-to-consumer telemedicine existed, payment prior to 2020 did not allow for feasible integration of telemedicine within US pediatric primary care practices, resulting in limited use in this setting [1,2]. Changes during the pandemic-related public health emergency in the United States [3] and the world [4] reduced technical barriers and supported payment to primary care clinicians providing telemedicine to patients at home, resulting in substantial rapid uptake in the use of telemedicine within pediatric primary care [2,5,6].

During the pandemic, key studies have examined use of telemedicine for pediatric primary care services. In the early pandemic, telemedicine comprised 44% of acute visits occurring in pediatric primary care practices in one analysis of commercial claims, with this falling to 15% of visits by the seventh month of the pandemic [2]. Using electronic health record data from April 2020, differential telemedicine use within primary care by child age, race/ethnicity, and insurance was reported for practice groups in North Carolina [7]. Also using electronic health record data from April 2020, rates of telemedicine use by patients of a primary care practice network in Pennsylvania differed by child age and race/ethnicity, but not by child insurance [6]. Additional work describes lower odds of telemedicine scheduling or use (relative to in-person visit scheduling or use) during the pandemic for children identified as Black, children with non–English speaking parents, and children who are Medicaid beneficiaries [8-10]. These analyses highlight the need to focus on potential factors contributing to inequities in telemedicine use as telemedicine use continues to evolve.

While prior studies have identified disparities in use of telemedicine within primary care through analysis of administrative or electronic health record data, these analyses focus on clinical (rather than community) populations and on the limited variables available in administrative data [6,7,10-12]. To complement these prior studies, we surveyed a nationally representative sample of parents to investigate parent-reported use of primary care-based telemedicine across sociodemographic factors as well as parent-reported measures of technology access, health literacy, self-efficacy, and social support. Given known disparities in access to primary care [13], we limited our analysis of primary care telemedicine to those with a usual source of care for their children. Thus, we aimed to identify factors associated with differential use of primary care telemedicine by children among families reporting a usual source of pediatric care.

## Methods

### Ethical Considerations

The University of Pittsburgh and the National Opinion Research Center (NORC) at the University of Chicago Institutional Review Boards determined this study to be exempt from human-subject review (STUDY21070080).

### Survey Development

We surveyed parents of children ≤17 years old on a nationally representative panel about health care–seeking for their children. The survey included items regarding prior telemedicine visits by the respondent’s child or children, as well as respondent demographics, access to technology, and measures of self-efficacy and health literacy. This was part of a larger survey with items regarding priorities and expectations when seeking care for children (Multimedia Appendix 1). The primary outcome of interest in this analysis was parent-reported primary care telemedicine visits by their child or children. Primary care telemedicine was defined within the survey as “a telemedicine visit with your child’s usual primary care office or clinic” with further elaboration that “this would be a virtual visit with the provider or providers that conduct in-person well and sick care for your child(ren) in the office or clinic.” Parents were asked first if they had ever used telemedicine for a visit for their child or children. Those indicating prior telemedicine use for their child or children were then asked where those prior telemedicine visits had occurred, with multiple answers accepted. Respondents endorsing a telemedicine visit “with my child’s usual primary care doctor or another doctor or staff member from my child’s usual doctor’s office” were considered as having experienced primary care telemedicine for a child. Other possible responses included “with a doctor from a direct-to-consumer telemedicine company,” “with a doctor at an urgent care or emergency department,” “with a specialist doctor (such as a surgeon, cardiologist) who my child could also see in-person,” “with a therapist, counselor, or mental health provider who my child could also see in-person,” “unsure/I don’t remember,” and “other.”

Respondents were also asked about their own, their child or children’s, and their family’s demographics, including their age, gender, educational attainment, income, and race/ethnicity; number of children and adults in the household; age of the youngest child; child insurance type; presence of a child with a chronic medical condition in the household; primary residence census region; and rural/urban status. We asked about technology access in terms of internet service and devices using questions from the American Community Survey [14], and we also inquired about potential internet scarcity or unreliability using investigator-generated items.

We asked about health literacy, self-efficacy, and social support due to potential associations with care-seeking decisions [15,16]. Health literacy was assessed using the single-item health literacy scale [17,18], which asked about respondents’ confidence in completing medical forms. We adapted the General Self Efficacy Scale [19] to the context of seeking health care for a child, and we also inquired who, if anyone, helped the respondent make health decisions for their child. Additional survey metadata detailed whether the survey was administered in English or Spanish and whether the family was surveyed via household internet or not.

We identified respondents with a usual source of care for their children based on 2 items, which asked whether there was (1) one place where they took their child or children for care and (2) one person who they thought of as their child or children’s regular doctor or nurse, consistent with items in the Parent’s Perception of Primary Care instrument [20]. Respondents affirming either of these items were considered to have a usual source of care for their child or children.

After development, we completed cognitive interviews with 3 parents of young children, with revisions made based on feedback. The survey was professionally translated into Spanish by the NORC team. The English and Spanish surveys were programmed into NORC’s electronic survey tool and piloted with 70 respondents; it was then refined further based on a review of responses (eg, additional response options were added based on write-in responses).

### Survey Administration

The finalized survey was then administered from February 17 to 28, 2022, through the AmeriSpeak panel, a probability-based panel designed to be representative of the US household population funded and operated by NORC at the University of Chicago [21]. The AmeriSpeak panel consists of randomly sampled US households contacted by US mail, telephone, and face-to-face field interviews. Based on preferences identified at panel enrollment, AmeriSpeak households participate in either English or Spanish and by either internet or telephone. Eligibility criteria for the current survey included being the parent or guardian of at least one child aged 17 years or younger in the household and being responsible for making medical decisions for at least one child in the household. Of 6015 panelists invited to participate, 27% (1599) completed the screener, with 81% (1297) of those screened determined to be eligible. Of those eligible, 93% completed the survey for a final sample of 1206. Respondents were excluded from these counts (n=64) if they were identified as completing the survey in less than one third of the median time of 17 minutes, refusing or skipping more than 50% of items, or submitting straight-lined responses to grid questions. Statistical weighting of responses occurred through computation of panel base sampling weights and study-specific sampling weights raked to external US population demographics from the Current Population Survey [22] to produce the final study weights applied throughout analysis. Regarding sample size, this is a secondary analysis of survey data originally designed and sized to estimate population proportions for a separate primary analysis still underway. Respondents received the cash equivalent of US $5 for completing the survey (for an equivalent of US $17.50/hour).

### Survey Analysis

We first compared sociodemographic characteristics of respondents who did and did not report a usual source of care for their children. We then examined factors associated with use of primary care telemedicine among those reporting a usual source of care for their children. Specifically, we used Rao-Scott *F* tests, which account for weighted survey data, to compare sociodemographic characteristics and child telemedicine visits of respondents with and without a usual source of care for their children [23,24]. Focusing specifically among those with a usual source of care for their children, we then used Rao-Scott *F* tests and 2-tailed *t* statistics from weighted linear regression models to compare respondent and family sociodemographic characteristics, technology access, and self-efficacy measures for respondents with children who had and had not used primary care telemedicine. Analyses were conducted using Stata (version 17; StataCorp), accounting for survey design and survey weights. Significance testing was performed using an α level of .05.

## Results

Of 1206 respondents, 312 (weighted percentage 27%) reported income less than US $30,000, 375 (weighted percentage 22%) identified as Hispanic, and 460 (weighted percentage 37%) reported their children’s insurance to be Medicaid or other federally funded insurance. A weighted percentage of 4% (70/1206) completed the survey in Spanish. Out of these 1206 respondents, 1054 (weighted percentage 87%) reported having a usual source of care for their children. Those with and without a usual source of pediatric care did not differ significantly by gender, survey language, household structure, metropolitan residence, or census region (Table 1). Respondents reporting a usual source of pediatric care were more likely, however, to be older, to have attained a higher educational level, to have higher household income, to identify as White, to have a young child in the house, and to have a child with a chronic medical condition. A weighted percentage of approximately 18% (200/1054) of those with a usual source of pediatric care reported a child with a chronic medical condition and a weighted percentage of 37% (404/1054) reported their child or children were beneficiaries of Medicaid or other federally funded health insurance.

Those with a usual source of pediatric care were more likely to report any telemedicine visits among their children (471/1054, weighted percentage 43% vs 45/152, 26%; *P*<.001) and any primary care telemedicine visits (301/1054, weighted percentage 28% vs 18/152, 12%; *P*<.001, Table 2) than those without a usual source of care. Other telemedicine visit types did not differ significantly between the groups.

**Table 1:** Respondents with and without a usual source of pediatric care. Weighted percentages and statistical testing accounted for survey design and survey weights. Statistical testing was performed using Rao-Scott *F* tests.

Characteristics		With usual source of pediatric care (n=1054), n (weighted %)	Without a usual source of pediatric care (n=152), n (weighted %)	Design-based *P* value	
**Respondent age (years)**					.045
	18-29	125 (10.9)	33 (18.1)		
	30-44	675 (59.6)	86 (56.7)		
	45-59	226 (26.5)	32 (25.1)		
	≥60	28 (3)	1 (0.1)		
**Respondent gender**					.11
	Male	353 (43.5)	67 (52.6)		
	Female	701 (56.5)	85 (47.4)		
**Educational attainment**					.002
	Less than high school	47 (7.3)	23 (19.5)		
	High school graduate or equivalent	142 (23.6)	29 (28.3)		
	Some college or vocational school	401 (25.9)	47 (19.4)		
	Bachelor’s degree	282 (26.6)	28 (17.6)		
	Postgraduate study/professional degree	182 (16.6)	25 (15.2)		
**Household income**					.005
	Less than US $30,000	265 (24.8)	57 (42.5)		
	US $30,000 to under US $60,000	262 (23.5)	45 (20.2)		
	US $60,000 to under US $100,000	271 (25.1)	31 (20.5)		
	US $100,000 or more	256 (26.5)	19 (16.9)		
**Respondent race/ethnicity**					.03
	Asian, non-Hispanic	26 (5.9)	8 (9.7)		
	Black, non-Hispanic	90 (10.7)	19 (15.2)		
	Hispanic	312 (20.6)	63 (31.3)		
	Other, non-Hispanic	47 (3.9)	7 (2.8)		
	White, non-Hispanic	579 (59)	55 (41.1)		
**Survey language**					.40
	English	995 (96.3)	141 (94.6)		
	Spanish	59 (3.7)	11 (5.4)		
**Number of children in household^a^**					.07
	1	360 (32.2)	63 (36.5)		
	2	412 (37)	62 (44.7)		
	≥3	282 (30.8)	26 (18.8)		
**Number of adults in household**					.42
	1	166 (14.9)	28 (18.2)		
	≥2	888 (85.1)	124 (81.8)		
**Age of youngest child (years)**					<.001
	0-1	180 (16.8)	21 (13.9)		
	2-5	323 (30.4)	30 (17.4)		
	6-11	313 (30.7)	36 (26.1)		
	12-17	238 (22.1)	65 (42.6)		
**Child insurance type^a^**					<.001
	Through employer or purchased directly	632 (61.8)	75 (49.3)		
	Medicaid or other federal payer	404 (36.7)	56 (39.8)		
	Uninsured	15 (1.6)	17 (10.9)		
**Child with chronic medical condition^a^**					<.001
	Yes	200 (18.3)	14 (9.7)		
	No	821 (78.5)	113 (73.9)		
	Unsure	31 (3.2)	22 (16.4)		
**Census region**					.20
	Northeast	149 (16.7)	13 (9.7)		
	Midwest	246 (21.3)	28 (17.4)		
	South	381 (37.6)	65 (46.6)		
	West	278 (24.4)	46 (26.4)		
**Metropolitan area**					.67
	Nonmetropolitan area	194 (19.3)	20 (17.3)		
	Metropolitan area	860 (80.7)	132 (82.7)		

^a^For these items, one or more respondents skipped the indicated item, such that respondents will sum to less than 1206.

**Table 2:** Telemedicine visits by children of respondents with and without a usual source of pediatric care. Weighted percentages and statistical testing accounted for survey design and survey weights. Statistical testing was performed using Rao-Scott *F* tests.

	With usual source of pediatric care (n=1054), n (weighted %)	Without usual source of pediatric care (n=152), n (weighted %)	*P* value
Telemedicine visit to any site	471 (43)	45 (26.3)	<.001
Primary care telemedicine visit	301 (27.8)	18 (12.2)	.001
Direct-to-consumer telemedicine visit	76 (6.7)	14 (8.9)	.45
Emergency department or urgent care telemedicine visit	34 (3.3)	8 (3.7)	.86
Specialist telemedicine visit	72 (5.7)	5 (3.4)	.39
Mental health telemedicine visit	86 (8)	2 (1.7)	.06

Among those with a usual source of pediatric care, family and child demographic factors associated with increased likelihood of primary care telemedicine visits for children included having a child with a chronic medical condition (87/301, weighted percentage 27% of those with vs 113/753, weighted percentage 15% of those without primary care telemedicine use; *P*=.002) and residing in a metropolitan area (262/301, weighted percentage 88% of those with vs 598/753, weighted percentage 78% of those without primary care telemedicine use; *P*=.004, Table 3). Respondent age and gender were also associated with increased reported use of primary care telemedicine. Among those with a usual source of pediatric care, reported use of primary care telemedicine for children did not vary by other examined demographic factors, such as respondent educational attainment, income, race/ethnicity, or household composition.

Specific internet service plan type and device ownership were not significantly associated with primary care telemedicine use (Table 4), with the exception that a higher percentage of those without primary care telemedicine use (8/753, 1%) reported no device ownership compared to those with primary care telemedicine use (1/301, 0.1%; *P*=.009). Those reporting primary care telemedicine use were more likely to affirm that they worried about internet access scarcity or unreliability (60/301, 24% of those with vs 116/753, 16% of those without primary care telemedicine use; *P*=.05).

Those with high health literacy comprised a weighted percentage of 96% (285/301) of primary care telemedicine users compared to a weighted percentage of 91% (693/753) of primary care telemedicine nonusers (*P*=.005, Table 5). Self-efficacy at care-seeking for children did not vary significantly by primary care telemedicine use, nor did involvement of others in child health decisions.

**Table 3:** Use of primary care telemedicine among respondents with a usual source of pediatric care by family and child demographics. Weighted percentages and statistical testing accounted for survey design and survey weights. Statistical testing was performed using Rao-Scott *F* tests.

Characteristics		Child with PCP^a^ telemedicine use (n=301), n (weighted %)	Child without PCP telemedicine use (n=753), n (weighted %)	Design-based *P* value	
**Respondent age (years)**					.03
	18-29	30 (6.3)	95 (12.6)		
	30-44	208 (64.7)	467 (57.6)		
	45-59	56 (23.6)	170 (27.7)		
	≥60	7 (5.4)	21 (2.1)		
**Respondent gender**					.02
	Male	88 (35.3)	265 (46.6)		
	Female	213 (64.7)	488 (53.4)		
**Educational attainment**					.75
	Less than high school	14 (9)	33 (6.6)		
	High school graduate or equivalent	35 (20.7)	107 (24.7)		
	Vocational/technical/some college/associate degree	119 (27.7)	282 (25.2)		
	Bachelor’s degree	78 (25.5)	204 (27)		
	Postgraduate study/professional degree	55 (17.1)	127 (16.5)		
**Household income**					.81
	Less than US $30,000	73 (24.6)	192 (24.9)		
	US $30,000 to under US $60,000	78 (22.9)	184 (23.7)		
	US $60,000 to under US $100,000	79 (27.4)	192 (24.1)		
	US $100,000 or more	71 (24.6)	185 (27.3)		
**Race/ethnicity**					.72
	Asian, non-Hispanic	7 (6.8)	19 (5.6)		
	Black, non-Hispanic	27 (12.4)	63 (10)		
	Hispanic	93 (18.9)	219 (21.2)		
	Other, non-Hispanic	15 (5)	32 (3.4)		
	White, non-Hispanic	159 (56.8)	420 (59.8)		
**Survey language**					.99
	English	282 (96.3)	713 (96.3)		
	Spanish	19 (3.7)	40 (3.7)		
**Number of children in household**					.14
	1	89 (25)	271 (35)		
	2	130 (39.4)	282 (36.1)		
	≥3	82 (35.6)	200 (28.9)		
**Number of adults in household**					.29
	1	56 (18.8)	110 (13.4)		
	≥2	145 (81.2)	643 (86.6)		
**Age of youngest child (years)**					.96
	0-1	50 (16.7)	130 (16.9)		
	2-5	90 (29)	233 (31)		
	6-11	96 (31)	217 (30.6)		
	12-17	65 (23.4)	173 (21.6)		
**Child insurance type^b^**					.17
	Through employer or purchased directly	176 (61)	456 (62.1)		
	Medicaid or other federal payer	124 (39)	280 (35.8)		
	Uninsured	0 (0)	15 (2.2)		
**Children with chronic medical conditions^b^**					.002
	Yes	87 (27.1)	113 (14.9)		
	No	203 (69.9)	618 (81.8)		
	Unsure	10 (3)	21 (3.3)		
**Census region**					.08
	Northeast	51 (20.2)	98 (15.3)		
	Midwest	68 (22.6)	178 (20.9)		
	South	87 (29.1)	294 (40.8)		
	West	95 (28.1)	183 (23)		
**Metropolitan area**					.004
	Nonmetropolitan Area	39 (12.3)	155 (22)		
	Metropolitan Area	262 (87.7)	598 (78)		

^a^PCP: primary care provider.

^b^For these items, one or more respondent skipped the item, such that respondents will not add to 1054.

**Table 4:** Use of primary care telemedicine among respondents with a usual source of pediatric care by technology access. Weighted percentages and statistical testing accounted for survey design and survey weights. Statistical testing was performed using Rao-Scott *F* tests.

			Child with PCP^a^ telemedicine use (n=301), n (weighted %)		Child without PCP telemedicine use (n=753), n (weighted %)		Design-based *P* value
**Survey modality**							.32
	Noninternet survey household	31 (9.3)		86 (12.3)			
	Internet survey household	270 (90.7)		667 (87.7)			
**Type of internet service (multiple allowed)^b^**							
	Cellular data plan for a mobile device	272 (89.3)		653 (86.9)		.48	
	Broadband (high speed) internet service	244 (81.5)		609 (81.7)		.95	
	Satellite, dial-up, or some other connection	18 (4.4)		45 (5)		.71	
	Cellular plan and no other internet service	42 (14.9)		98 (12.8)		.51	
	None of these	1 (0.1)		5 (0.6)		.28	
**Type of device (multiple allowed)^b^**							
	Desktop or laptop	239 (79.6)		611 (78.6)		.80	
	Smartphone	275 (90.7)		693 (91.4)		.78	
	Tablet or other portable computer	186 (60.6)		486 (63.7)		.50	
	Smartphone and no other device	43 (13.7)		90 (13.7)		.99	
	None of these	1 (0.1)		8 (1)		.009	
**Internet connectivity concerns**							
	Always or often worried about internet access or reliability	60 (23.9)		116 (16.4)		.05	

^a^PCP: primary care provider.

^b^For these items, one or more respondent skipped the item, such that respondents will sum to less than 1054.

**Table 5:** Among children with a usual place for medical care, use of primary-care-provider telemedicine by health literacy and self efficacy. Weighted percentages and statistical testing accounted for survey design and survey weights. Statistical testing was performed using Rao-Scott *F* tests and *t* statistics from weighted linear regression models.

	Child with PCP^a^ telemedicine use (n=301)	Child without PCP telemedicine use (n=753)	Design-based *P* value
Confident filling out medical forms alone, n (weighted %)	285 (96.2)	693 (90.5)	.005
Total General Self-Efficacy Score, mean (SD)	19.0 (2.8)	18.7 (2.9)	.27
No one else involved in health decisions for child, n (weighted %)	52 (16.9)	101 (11.4)	.09

^a^PCP: primary care provider.

## Discussion

In a national sample of respondents with a usual source of care for their children, over one-quarter reported use of primary care telemedicine for their children as of 2022. Reported pediatric primary care telemedicine use was higher for respondents with children with chronic conditions, residence in a metropolitan area, higher health literacy, and—surprisingly—higher worry about internet connectivity.

Increased likelihood of telemedicine use for those with children with chronic conditions may be expected due to increased underlying need for or use of medical care or increased desire to reduce exposure to illness. Child primary care telemedicine use also varied with respondent age and gender, but otherwise did not vary with key demographics often associated with disparate health care use, including respondent race/ethnicity, educational attainment, or income. This provides reason for optimism regarding telemedicine’s potential to be used equitably within primary care practices among patients with a usual source of care. However, there are substantial inequities in the characteristics of respondents who reported that their children did versus did not have a usual source of primary care across educational attainment, income, child insurance, and race/ethnicity, echoing inequities in having a usual source of care in prior studies [25-29]. Together, these findings indicate that in order to advance equitable use of primary care telemedicine across these sociodemographic characteristics, the largest impact may be through ensuring equitable access to a usual source of pediatric care.

Our finding of increased primary care telemedicine use among those residing in metropolitan areas is similar to prior examinations of other telemedicine modalities [30,31]. Interestingly, differential internet connectivity is often suggested as a potential explanation for rural/urban disparities in telemedicine use, but we observed similar rates of primary care telemedicine use across categories reflecting device ownership, internet service, and survey modality. An alternative explanation for lower primary care telemedicine use among nonmetropolitan residents that warrants exploration could be differential ability to offer and sustain telemedicine in rural primary care practices, perhaps due to technology barriers or workforce capacity [32,33].

As for the lack of significant difference in devices and internet between primary care telemedicine users and nonusers, an optimistic interpretation of this finding could be that in the context of a primary care relationship, clinicians and practices work to collaboratively overcome device or technology barriers with patients in advance or at the point of care. Data from specific health systems do indeed show that primary care interventions (eg, having medical assistants virtually room patients) can reduce disparities in no-show rates for scheduled telemedicine visits [34]. Our analysis yielded yet another surprising finding about connectivity in that our items to examine internet scarcity identified greater worry about internet connectivity and reliability among primary care telemedicine users than nonusers. This could be due to actual increased use of this modality of telemedicine among those with more tenuous internet connections (perhaps due to other constraints, such as transportation or time leading to telemedicine use) or could be due to those who have used telemedicine having more experiences of connectivity problems (or greater recall of such experiences). Of note, these items asked about internet connectivity in general and not specifically in health care contexts. Together, these results illustrate the complexity and importance of measuring different components of the digital divide.

While self-efficacy and the support of others in child health decisions could influence care-seeking [15,16], we found that this was not the case. We did, however, find that higher health literacy was associated with increased telemedicine use. Whether this reflected greater ability to self-advocate for virtual care when desired, greater confidence in the ability to communicate the children’s needs virtually, or differential triage by office staff is not clear and should be evaluated as telemedicine practices evolve.

Looking toward the future, these data suggest that within pediatric primary care, there are key opportunities to maintain and improve equity in telemedicine use. First, lower use of telemedicine among those with lower health literacy and in nonmetropolitan communities highlights opportunities to ensure that messaging about telemedicine is designed for lower health-literacy populations, that scheduling and connection processes are designed for usability by these populations, and that rural practices and communities have the infrastructure they need to support telemedicine at the practice and community level. Second, maintaining financial access to telemedicine within pediatric primary care practices is imperative to continuing this model of care, which requires state Medicaid and commercial payers to maintain payment for these telemedicine visits when primary care pediatricians connect with patients at home. Incentives outside of fee-for-service payments may also be necessary to encourage practices to continue offering telemedicine, such as recognition of the value of telemedicine for patients within medical home certification or value-based payment arrangements. Third, higher use among children with chronic medical conditions suggests acceptability among families and caregivers; expansion of primary care telehealth, perhaps coupled with expanded remote patient monitoring, may enhance receipt of care while reducing family burden. Fourth, because access to primary care telemedicine requires a usual source of care, efforts to ensure children have financial and physical access to primary care medical homes is required for ongoing access to primary care telemedicine. And because use of telemedicine within pediatric primary care will continue to evolve in ways where biases and barriers may become more prominent after the early pandemic period, we must continue close evaluation of opportunities to enhance access, outcomes, and equity through timely evaluation in the coming months and years.

Our analysis focused on any primary care telemedicine use by the respondents’ children and did not assess quality or frequency of telemedicine use within primary care. Thus, while we did not find significant differences in use of telemedicine for many sociodemographic factors, subtler inequities within primary care telemedicine use may remain. For example, we note that we asked about “ever” using primary care telemedicine and that we fielded this survey 2 years into the COVID-19 related public health emergency (in February 2022). As a result, some respondents may have reported on primary care telemedicine use that occurred specifically during the early pandemic, when a wider range of applications was being used to facilitate connection. Whether differences in primary care telemedicine use by device and internet service might be detected during specific later time frames warrants further evaluation, as access could be more limited or inequitable when specific applications are required for connection and if resumption of in-person capacity introduces greater biases in triage practices. We also did not assess unmet needs for care generally or for telemedicine specifically. If underlying need for care is different across demographic groups, then similar rates of telemedicine use may still leave inequities in unmet need. An additional limitation is that our data come from self-report by parents reporting for the children in their family rather than for individual children, although responses did not differ significantly for families with more children. We note that we did not ask for details regarding the site of usual care or telemedicine availability at that site. Additionally, because we did not include individuals without a usual source of care for their children in our analysis of primary care telemedicine use, we wish to emphasize that our results would not be expected to generalize beyond those with a usual source of care.

In conclusion, among respondents whose children have a usual source of care, approximately one-quarter reported use of primary care telemedicine for their children as of 2022. Compared to respondents who had not used primary care telemedicine for their children, primary care telemedicine users were more likely to have higher parental health literacy, to reside in metropolitan areas, and to report worry about their internet connectivity, but were similar for other sociodemographic variables. Equitable access to primary care telemedicine may be enhanced by promoting access to primary care for children, sustaining payment for primary care telemedicine, addressing barriers in nonmetropolitan practices and communities, and designing for lower health-literacy populations.

## Appendix

Multimedia Appendix 1 Survey instrument.

## Abbreviations

NORC: National Opinion Research Center
PCP: primary care provider
USOC: usual source of care
