# Supplementary material for: Parent-Reported Use of Pediatric Primary Care Telemedicine: Survey Study
Source: J Med Internet Res. 2023 Feb 9;25:e42892. doi: 10.2196/42892 (PMC9951070; doi:10.2196/42892)
Supplement: Multimedia Appendix 1 [file jmir_v25i1e42892_app1.docx]

**Children And Colds Telemedicine Use Survey**

**Fielded February 2022**

**Screening Questions:**

Are you the parent or guardian of at least one child ages 0-17 years old in your household?

RESPONSE OPTIONS:

1. Yes
2. No

Are you responsible for making medical decisions for at least one child ages 0-17 years old in your household?

RESPONSE OPTIONS:

1. Yes
2. No

How many children aged 0-17 live in your household?

Please tell us the age of the [child/children] living in your household for whom you are the parent or legal guardian, starting with the youngest.

This survey is part of a research study being conducted by scientists at the University of Pittsburgh.

In this survey we will be asking how families make decisions about seeking care for their children when those children are sick with cold symptoms.

Our goal is to use your answers to help improve healthcare for all families with children.

We will ask you to think about how you make decisions about where you seek care for your child.

We will also ask a few brief questions about you and your background. *If any questions make you feel uncomfortable, you may skip any questions you do not wish to answer*. However, your thoughts are very important to us.

This is a confidential survey, and your answers will be securely stored separate from your contact information. Your participation is voluntary, and you may stop the survey at any time.

The following questions ask about the things that are important to you when you are deciding where to seek care for your sick child(ren). Please read the text of the question carefully before responding.

Imagine that **your child(ren) are sick with a cold** (such as a cough and runny nose). You decide that they need to be seen by a health care provider (e.g., doctor, nurse practitioner, physician assistant), and you're considering **where to take your child(ren)**: to their usual primary care practice, an urgent care, an emergency department, or for a telemedicine visit.

If you have multiple children and feel that your answers would be different for different children, please think about your youngest child as you answer.

Please tell us how important each of the following things are to you when deciding where to seek care for your child(ren) when they have cough and cold like symptoms.

GRID ITEMS

1. Getting to see a provider who knows my child (my child’s “usual” provider, a "familiar face")
2. Getting to see a provider with experience caring for children
3. Getting to see a provider who has full access to my child’s medical history or medical records
4. Getting care in a way that protects my child’s privacy
5. Getting care where the provider will be able to do all of tasks and tests that might be needed to care for my child
6. Getting care from a provider who I trust to make choices in my child’s best interest
7. Getting care at a place that doesn’t disrupt my regular schedule
8. Getting care at a place where I won't have to pay a lot
9. Getting care at a place that doesn’t involve a lot of travel time and/or hassle
10. Getting care at a place that is clean and well-maintained
11. Getting care at a place where my children aren’t near other sick children
12. Getting care at a place where I can easily get a medical excuse, prescription, or other document from the doctor
13. Getting care at a place where I don’t have to wait for a long time be seen
14. How bad my child’s symptoms are
15. How long my child has been sick
16. The number of different symptoms my child has
17. My child’s medical history (previous illnesses or health complications)
18. How worried I am that my child might have a serious illness
19. How my child’s mood and energy levels are
20. How easily I think I will be able to show or explain what is wrong with my child
21. Where my child will be comfortable and able to cooperate
22. How young my child is

RESPONSE OPTIONS:

1. Not a priority for this decision
2. Low priority for this decision
3. Medium priority for this decision
4. High priority for this decision
5. Essential priority for this decision

Health care providers provide care for children’s cough and cold like symptoms at different types of locations. Some work at pediatric or primary care offices, some work in urgent care, some work in emergency rooms, and some will treat children over telemedicine, using a computer or phone. Now we want to understand what you might expect to find at these different care locations.

Imagining these different locations, please tell us how likely you think you will find each of the following things at the different places where your child might receive care.

These next questions ask about how likely it is that you will find each of the following when visiting your child(ren)’s primary care office or clinic in-person. A primary care office or clinic is where your child(ren) are seen for a routine checkup or non-emergency medical care.

When I visit my child(ren)’s usual primary care office or clinic in-person for cough and cold like symptoms, I imagine I will find…

GRID ITEMS:

1. A provider who knows my child (my child’s “usual” provider, a "familiar face")
2. A provider with experience caring for children
3. A provider with full access to their medical history or medical records
4. Care that protects my child’s privacy
5. A provider who will be able to do all of tasks and tests that might be needed to care for my child
6. A provider who I trust to make choices in my child’s best interest
7. A provider who will consider my child’s medical history (previous illnesses or health complications)
8. A provider who will be able to provide care if my child’s symptoms are bad or severe
9. A provider who will be able to provide care if my child has been sick for a long time
10. A provider who will be able to provide care if my child has a number of different symptoms
11. A provider who will be able to provide care if my child might have a serious illness
12. A provider who will be able to provide care if my child’s mood and energy levels are low
13. A provider who will understand when I explain what is wrong with my child
14. A provider who my child will be comfortable and able to cooperate with
15. A provider who will provide care across all ages 0-17 years old
16. A visit that does not disrupt my regular schedule
17. A visit where I won't have to pay a lot
18. A visit that doesn’t involve a lot of travel time and/or hassle
19. A visit at a place that is clean and well-maintained
20. A visit where my children aren’t near other sick children
21. A visit where I can easily get a medical excuse, prescription, or other document from the doctor
22. A visit where I don’t have to wait for a long time to be seen

RESPONSE OPTIONS:

1. Never
2. Rarely
3. Sometimes
4. Often
5. Always

These next questions ask about how likely it is that you will find each of the following when visiting an urgent care office in-person. An urgent care office is a walk-in clinic for treating minor illnesses and injuries that is often open after hours. It may be located within a larger hospital or hospital system or may be a free-standing clinic.

When I visit an urgent care office in-person for my child(ren)'s cough and cold like symptoms, I imagine I will find…

GRID ITEMS:

1. A provider who knows my child (my child’s “usual” provider, a "familiar face")
2. A provider with experience caring for children
3. A provider with full access to their medical history or medical records
4. Care that protects my child’s privacy
5. A provider who will be able to do all of tasks and tests that might be needed to care for my child
6. A provider who I trust to make choices in my child’s best interest
7. A provider who will consider my child’s medical history (previous illnesses or health complications)
8. A provider who will be able to provide care if my child’s symptoms are bad or severe
9. A provider who will be able to provide care if my child has been sick for a long time
10. A provider who will be able to provide care if my child has a number of different symptoms
11. A provider who will be able to provide care if my child might have a serious illness
12. A provider who will be able to provide care if my child’s mood and energy levels are low
13. A provider who will understand when I explain what is wrong with my child
14. A provider who my child will be comfortable and able to cooperate with
15. A provider who will provide care across all ages 0-17 years old
16. A visit that does not disrupt my regular schedule
17. A visit where I won't have to pay a lot
18. A visit that doesn’t involve a lot of travel time and/or hassle
19. A visit at a place that is clean and well-maintained
20. A visit where my children aren’t near other sick children
21. A visit where I can easily get a medical excuse, prescription, or other document from the doctor
22. A visit where I don’t have to wait for a long time to be seen

RESPONSE OPTIONS:

1. Never
2. Rarely
3. Sometimes
4. Often
5. Always

These next questions ask about how likely it is that you will find each of the following when visiting an emergency department. An emergency department provides medical care to patients as they arrive and is capable of stabilizing critically ill patients. It may exist within a larger hospital or hospital system or may be a free-standing emergency department.

When I visit an emergency department in-person for my child(ren)'s cough and cold like symptoms, I imagine I will find…

GRID ITEMS:

1. A provider who knows my child (my child’s “usual” provider, a "familiar face")
2. A provider with experience caring for children
3. A provider with full access to their medical history or medical records
4. Care that protects my child’s privacy
5. A provider who will be able to do all of tasks and tests that might be needed to care for my child
6. A provider who I trust to make choices in my child’s best interest
7. A provider who will consider my child’s medical history (previous illnesses or health complications)
8. A provider who will be able to provide care if my child’s symptoms are bad or severe
9. A provider who will be able to provide care if my child has been sick for a long time
10. A provider who will be able to provide care if my child has a number of different symptoms
11. A provider who will be able to provide care if my child might have a serious illness
12. A provider who will be able to provide care if my child’s mood and energy levels are low
13. A provider who will understand when I explain what is wrong with my child
14. A provider who my child will be comfortable and able to cooperate with
15. A provider who will provide care across all ages 0-17 years old
16. A visit that does not disrupt my regular schedule
17. A visit where I won't have to pay a lot
18. A visit that doesn’t involve a lot of travel time and/or hassle
19. A visit at a place that is clean and well-maintained
20. A visit where my children aren’t near other sick children
21. A visit where I can easily get a medical excuse, prescription, or other document from the doctor
22. A visit where I don’t have to wait for a long time to be seen

RESPONSE OPTIONS:

1. Never
2. Rarely
3. Sometimes
4. Often
5. Always

These next questions ask about how likely it is that you will find each of the following during a telemedicine visit with your child's usual primary care office or clinic.  This would be a virtual visit with the provider or group of providers that conduct in-person well and sick care for your child(ren) in the office or clinic.

When I have a telemedicine visit with my child(ren)'s usual primary care office or clinic for cough and cold like symptoms, I imagine will find …

GRID ITEMS:

1. A provider who knows my child (my child’s “usual” provider, a "familiar face")
2. A provider with experience caring for children
3. A provider with full access to their medical history or medical records
4. Care that protects my child’s privacy
5. A provider who will be able to do all of tasks and tests that might be needed to care for my child
6. A provider who I trust to make choices in my child’s best interest
7. A provider who will consider my child’s medical history (previous illnesses or health complications)
8. A provider who will be able to provide care if my child’s symptoms are bad or severe
9. A provider who will be able to provide care if my child has been sick for a long time
10. A provider who will be able to provide care if my child has a number of different symptoms
11. A provider who will be able to provide care if my child might have a serious illness
12. A provider who will be able to provide care if my child’s mood and energy levels are low
13. A provider who will understand when I explain what is wrong with my child
14. A provider who my child will be comfortable and able to cooperate with
15. A provider who will provide care across all ages 0-17 years old
16. A visit that does not disrupt my regular schedule
17. A visit where I won't have to pay a lot
18. A visit that doesn’t involve a lot of travel time and/or hassle
19. A visit where my children aren’t near other sick children
20. A visit where I can easily get a medical excuse, prescription, or other document from the doctor
21. A visit where I don’t have to wait for a long time to be seen

RESPONSE OPTIONS:

1. Never
2. Rarely
3. Sometimes
4. Often
5. Always

These next questions ask about how likely it is that you will find each of the following when having a telemedicine visit with a telemedicine company or group that focuses on telemedicine visits rather than in-person care (also called direct-to-consumer telemedicine).  Providers in these groups do not provide care in-person and are not part of your child's usual care team.

In these visits, you connect online and see an available provider in a model that could be thought of as virtual urgent care. Some direct-to-consumer telemedicine groups or companies are affiliated with health systems but are still separate from primary care clinics who might see their own patients through telemedicine.

When I have a telemedicine visit with a direct-to-consumer telemedicine company for my child(ren)'s cough and cold like symptoms, I imagine will find …

GRID ITEMS:

1. A provider who knows my child (my child’s “usual” provider, a "familiar face")
2. A provider with experience caring for children
3. A provider with full access to their medical history or medical records
4. Care that protects my child’s privacy
5. A provider who will be able to do all of tasks and tests that might be needed to care for my child
6. A provider who I trust to make choices in my child’s best interest
7. A provider who will consider my child’s medical history (previous illnesses or health complications)
8. A provider who will be able to provide care if my child’s symptoms are bad or severe
9. A provider who will be able to provide care if my child has been sick for a long time
10. A provider who will be able to provide care if my child has a number of different symptoms
11. A provider who will be able to provide care if my child might have a serious illness
12. A provider who will be able to provide care if my child’s mood and energy levels are low
13. A provider who will understand when I explain what is wrong with my child
14. A provider who my child will be comfortable and able to cooperate with
15. A provider who will provide care across all ages 0-17 years old
16. A visit that does not disrupt my regular schedule
17. A visit where I won't have to pay a lot
18. A visit that doesn’t involve a lot of travel time and/or hassle
19. A visit where my children aren’t near other sick children
20. A visit where I can easily get a medical excuse, prescription, or other document from the doctor
21. A visit where I don’t have to wait for a long time to be seen

The next set of questions are about your use of technology. These questions will help us understand the different access to tools among our survey respondents.

Have you ever used telemedicine for a visit for your child(ren) in the past?

RESPONSE OPTIONS:

1. Yes, once
2. Yes, more than once
3. No
4. Unsure / I don't remember

Where was that telemedicine visit?

*Please select all that apply.*

RESPONSE OPTIONS:

1. With my child’s usual primary care doctor or another doctor or staff member from my child’s usual doctor’s office
2. With a doctor from a direct-to-consumer telemedicine company
3. With a doctor at an urgent care or emergency department
4. With a specialist doctor (such as a surgeon, cardiologist) who my child also could see in-person
5. With a therapist, counselor, or mental health provider who my child also could see in-person
6. Unsure / I don't remember
7. Other, please specify:

Have you used telemedicine for a visit for yourself in the past?

RESPONSE OPTIONS:

1. Yes, once
2. Yes, more than once
3. No
4. Unsure / I don't remember

Where was that telemedicine visit?

*Please select all that apply.*

RESPONSE OPTIONS:

1. With my usual primary care doctor or another doctor or staff member from my usual doctor’s office
2. With a doctor from a direct-to-consumer telemedicine company
3. With a doctor at an urgent care or emergency department
4. With a specialist doctor (such as a surgeon, cardiologist) who I could also see in-person
5. With a therapist, counselor, or mental health provider who I could also see in-person
6. Unsure / I don't remember
7. Other, please specify:

Do you or any member of your household own or use any of the following types of computers?

*Please select all that apply.*

RESPONSE OPTIONS:

1. Desktop or Laptop
2. Smartphone
3. Tablet or other portable computer
4. Some other type of computer, please specify:
5. None of these [

Do you or any member of this household have access to the internet using a:

*Please select all that apply.*

RESPONSE OPTIONS:

1. Cellular data plan for a smartphone or other mobile device
2. Broadband (high speed) internet service such as cable, fiber optic, or DSL service installed in this household
3. Satellite internet service installed in this household
4. Dial-up internet service installed in this household
5. Some other device, please specify:
6. None of these

Think about the adults in your household who are responsible for your child(ren), including yourself. Are any of these adults deaf or do any of these adults have serious difficult hearing?

RESPONSE OPTIONS:

- - - 1. Yes
      2. No

Think about the adults in your household who are responsible for your child(ren), including yourself. Are any of these adults blind or for any of these adults have serious difficulty seeing, even when wearing glasses?

RESPONSE OPTIONS:

- - - 1. Yes
      2. No

Think about the adults in your household who are responsible for your child(ren), including yourself. Because of a physical, mental, or emotional condition, do any of these adults have serious difficulty concentrating, remembering, or making decisions?

RESPONSE OPTIONS:

- - - 1. Yes
      2. No

Please tell us how often the following statements were true for you over the past 12-months.

In the last 12-months, I worried whether our internet access/data plan would run out or not be available when I really needed it.

RESPONSE OPTIONS:

1. Always True
2. Often True
3. Sometimes True
4. Never True

In the last 12-months, my internet connection was unreliable (such as too slow, poor sound, poor video quality, or video connection) when I really needed it.

RESPONSE OPTIONS:

1. Always True
2. Often True
3. Sometimes True
4. Never True

The next set of questions are about you and your child(ren)'s experiences with healthcare.

What type of health insurance do your child(ren) have?

*Please select all that apply.*

RESPONSE OPTIONS:

1. Insurance through a current or former employer or union
2. Insurance purchased directly from an insurance company
3. Medicaid, Medical Assistance, or another kind of government assistance plan for those with lower incomes or with a disability
4. TRICARE or other military healthcare
5. VA
6. Indian Health Service
7. Any other type of health insurance plan, please specify:
8. Uninsured

Does your family have a child(ren) who has a chronic health condition that results in the use of or need for more services (e.g., medical, mental health, educational) than is usual for most children of the same age?

RESPONSE OPTIONS:

1. Yes
2. No
3. Unsure / I don't know

Is there one particular place that you take your child(ren) for almost all their health care?

RESPONSE OPTIONS:

1. Yes
2. No
3. Unsure / I don't know

Is there one particular person that you think of as your child(ren)’s regular doctor or nurse?

RESPONSE OPTIONS:

1. Yes
2. No
3. Unsure / I don't know

Who else do you regularly involve in decision-making about your child’s health?

*Please select all that apply.*

RESPONSE OPTIONS:

1. Other parent whom I live with
2. Other parent whom I do not live with
3. Grandparent(s)
4. Other relative
5. Friends
6. Other, please specify:
7. No one other than myself

How confident are you filling out medical forms by yourself?

RESPONSE OPTIONS:

1. Extremely
2. Quite a bit
3. Somewhat
4. A little bit
5. Not at all

For the following items, think about how true they are when you are taking care of your children's health.

Please tell us the degree to which each of the following is true for you, ranging from “not at all true” to “exactly true” when taking care of your child(ren)'s health**.**

GRID ITEMS:

1. If someone opposes me , I can find the means and ways to get what I want.
2. It is easy for me to stick to my aims and accomplish my goals.
3. I am confident that I could deal efficiently with unexpected events.
4. I can remain calm when facing difficulties because I can rely on my coping abilities.
5. I can usually handle whatever comes my way.
6. Thanks to my resourcefulness, I know how to handle unforeseen situations.

RESPONSE OPTIONS:

1. Not at all true
2. Hardly true
3. Moderately true
4. Exactly true
